# Carbohydrates, Glycemic Index, and Glycemic Load in Relation to Bladder Cancer Risk

**DOI:** 10.3389/fonc.2020.530382

**Published:** 2020-09-23

**Authors:** Hejia Zhu, Qiwang Mo, Haixiang Shen, Song Wang, Ben Liu, Xin Xu

**Affiliations:** ^1^Department of Urology, The First Affiliated Hospital, Zhejiang University School of Medicine, Hangzhou, China; ^2^Department of Urology, Shenzhou People's Hospital, Shenzhou, China

**Keywords:** bladder cancer, carbohydrates, glycemic index, glycemic load, meta-analysis

## Abstract

**Objective:** Epidemiologic studies investigating the association between dietary carbohydrates as well as glycemic index and glycemic load (markers of carbohydrate quality) and bladder cancer risk have yielded inconsistent results. The aim of the present meta-analysis is to summarize the evidence on this association.

**Materials and Methods:** A comprehensive literature search of articles published by December 2019 was performed in PubMed, Scopus, and Web of Science databases. A random-effects model was used to calculate the pooled odds ratios (ORs) and their corresponding 95% confidence intervals (CIs).

**Results:** Twelve observational studies were included in the final analysis. There was no evidence of an association between consumption of carbohydrates and bladder cancer risk (pooled OR, 1.04; 95% CI, 0.92–1.17). No statistically significant association between glycemic load and bladder cancer was likewise found (pooled OR, 1.10; 95% CI, 0.85–1.42). However, there was a significant positive association between glycemic index and bladder cancer risk (pooled OR, 1.25; 95% CI, 1.11–1.41). In the dose–response analysis, the pooled OR (95% CI) per 10 units of glycemic index per day was 1.02 (95% CI, 1.01–1.04).

**Conclusion:** In this meta-analysis, glycemic index showed a positive linear association with bladder cancer risk.

## Introduction

Bladder cancer is a common human malignancy, with an estimated 429,800 new cancer cases and 165,100 deaths in 2012 worldwide (1). Despite recent improvement in diagnosis and therapy, the clinical outcome of bladder cancer remains poor, especially for muscle-invasive bladder cancer in elderly patients (2). Well-established risk factors for bladder cancer include tobacco smoking and exposure to certain chemicals in the working and general environments (3). Emerging evidence has shown that metabolic syndrome, obesity, low physical activity, and type 2 diabetes are related to an increased risk of bladder cancer (4–8), suggesting that insulin resistance may play a role in the etiology of bladder cancer.

Carbohydrates are one of the main dietary components affecting an individual's insulin secretion and blood glycemic response (9). Glycemic index is a measure of the blood glucose-raising potential of the carbohydrate-containing test food relative to a reference food, typically glucose, or white bread (10). Carbohydrate-containing foods can be classified as of high (≥70), moderate (56–69), or low (≤55) glycemic index relative to reference food (glycemic index = 100). Several studies have investigated the association between dietary carbohydrates as well as glycemic index and glycemic load (markers of carbohydrate quality) and bladder cancer risk; however, the results have been inconsistent. Overall, the previous studies consistently failed to find a statistically significant association between dietary carbohydrates and bladder cancer risk. Studies on glycemic index or glycemic load and bladder cancer risk have been mixed with null and positive associations (11–14). To clarify the association of intake of carbohydrates, glycemic index, and glycemic load within bladder cancer risk, we performed a systematic review and meta-analysis of all eligible studies.

## Methods

### Literature Search

A comprehensive literature search of articles published by December 2019 was performed in PubMed, Scopus, and Web of Science databases with the following search algorithm: (diet or dietary or nutrition or nutrient or food or glycemic index or glycemic load or glycemic index or glycemic load or carbohydrate or carbohydrates) and (tumor or cancer or carcinoma or neoplasm or malignancy) and (bladder or urinary tract or urothelial) and (cohort or case–cohort or case–control or case control). We also examined the reference lists from retrieved articles and other relevant reviews to identify any additional studies. This study was performed according to the Preferred Reporting Items for Systematic Reviews and Meta-Analyses statement (15).

### Inclusion Criteria

The inclusion criteria were as follows: (1) investigation of the association between dietary carbohydrates, glycemic index, or glycemic load and bladder cancer risk, (2) use of the following study design: case–control, nested case–control, cohort, and case–cohort studies as well as follow-up studies of randomized clinical trials, and (3) reporting of fully adjusted risk estimates, including relative risk (RR), hazard ratio (HR), or odds ratio (OR), and the corresponding 95% confidence intervals (CIs) for the association between bladder cancer and dietary carbohydrate, glycemic index, or glycemic load. If multiple publications used the same study population, the latest publication providing the largest sample size was included. The Participants, Intervention Comparators, Outcomes, Study Design criteria are presented in Supplementary Table 1.

### Data Extraction

The following data were extracted by two independent investigators (HZ and XX): first author's surname, publication year, country where the study was performed, specific characteristics of the study population, study design, age of participants, study size, number of cases, duration of follow-up for prospective studies, dietary assessment method, exposure (dietary carbohydrate, glycemic index, or glycemic load), quantity of intake, risk estimates, 95% CIs from the most fully adjusted models, and matched or adjusted variables (Table 1).

**Table 1:** Main characteristics of included studies in the meta-analysis.

**References**	**Year**	**Study region**	**Study design**	**Study population or source**	**No of cases**	**No of controls or cohort**	**Follow-up, year**	**Age, year**	**Methods of diet assessment**	**Types of exposure**	**Matched or adjusted variables**
Augustin et al. (11)	2017	Italy	Case-control	Within an established Italian network of collaborating centers	578	608	NA	Range: 25–84	Interviewer-administered FFQ	Carbohydrate, GI and GL	Age, sex, study center, education, smoking habits, alcohol drinking, abdominal obesity, and total energy
Sieri et al. (14)	2017	Italy	Prospective cohort	EPIC-Italy study	136	45,148	14.9	NR	Self-administered FFQ	GI and GL	Age, sex, education, smoking, BMI, alcohol intake, fiber intake, saturated fat intake, non-alcohol energy intake, and physical activity
Hu et al. (13)	2013	Canada	Case-control	NECSS	1,345	5,039	NA	Range: 20–76	Self-administered FFQ	GI and GL	Age, province, education, BMI, alcohol consumption, smoking, and energy intake
Allen et al. (16)	2013	Multiple Countries	Prospective cohort	EPIC	1,416	469,339	11.3	Median: 51	Self-administered FFQ	Carbohydrate	Age, sex, center, BMI, smoking, and total energy intake
George et al. (12) (Male cohort)	2009	USA	Prospective cohort	NIH-AARP diet and health study	857	262,642	6.16	Range: 50–71	Self-administered FFQ	GI and GL	Age, race/ethnicity, education, marital status, BMI, family history of any cancer, physical activity, smoking, alcohol consumption, and energy intake
George et al. (12) (Female cohort)	2009	USA	Prospective cohort	NIH-AARP diet and health study	322	183,535	6.16	Range: 50–71	Self-administered FFQ	GI and GL	Age, race/ethnicity, education, marital status, BMI, family history of any cancer, physical activity, smoking, alcohol consumption, and energy intake
Michaud et al. (17)	2000	USA	Prospective cohort	Health professionals follow-up study	320	47,909	12	Range: 40-75	Self-administered FFQ	Carbohydrate	Age, smoking, geographic region, total fluid intake, cruciferous vegetable intake, and energy intake
Wakai et al. (18)	2000	Japan	Case-control	A study from 1996 to 1999 in aichi prefecture, central Japan	297	295	NA	Mean: 66.6 (cases), 66.5 (controls)	Interviewer-administered FFQ	Carbohydrate	Age, gender, hospital, smoking, occupational history as a cook, and energy intake
Bruemmer et al. (19)	1996	USA	Case-control	SEER Program	262	405	NA	Range: 45–65	Self-administered FFQ	Carbohydrate	Age, gender, county, smoking, and energy intake
Chyou et al. (20)	1993	USA	Prospective cohort	A study conducted in Hawaii Japanese-American men	96	7,995	22	NR	Interviewer-administered FFQ	Carbohydrate	Age and smoking
Vena et al. (21)	1992	USA	Case-control	A study from 1979 to 1985 in western New York	351	855	NA	Range: 35-90	Interviewer-administered FFQ	Carbohydrate	Age, sex, neighborhood of residence, education, smoking, and energy intake
Riboli et al. (22)	1991	Spain	Case-control	A multi-center study from 1985 to 1986 in 5 regions of Spain	432	792	NA	Range: <80	Interviewer-administered FFQ	Carbohydrate	Age, sex, area of residence, smoking, and energy intake
Steineck et al. (23)	1990	Sweden	Case-control	A study from 1985 to 1987 in Stockholm	319	382	NA	NR	Self-administered FFQ	Carbohydrate	Age, sex, and smoking

*BMI, body mass index; No, number; EPIC, European Prospective Investigation into Cancer; FFQ, food-frequency questionnaire; GI, glycemic index; GL, glycemic load; NECSS, National Enhanced Cancer Surveillance System; NIH-AARP, National Institutes of Health-American Association of Retired Persons; SEER, Surveillance, Epidemiology, and End Results; NA, not applicable; NR, not reported*.

### Study Quality Assessment

The quality of each study was assessed by the same two investigators (HZ and XX) using the Newcastle–Ottawa Scale (NOS) (http://www.ohri.ca/programs/clinical_epidemiology/oxford.asp). Any disagreements were resolved by discussing with a third reviewer. NOS is an eight-item instrument that allows for the assessment of the study population and selection, study comparability, follow-up, and the outcome of interest. The range of possible scores is 0 to 9. Scores <7 and ≥7 were assigned for low- and high-quality studies, respectively.

### Statistical Methods

As the incidence of bladder cancer is relatively low, the RR and the HR were assumed to be approximately equivalent to the OR, and the OR was used as the study outcome. Associations between dietary factors (carbohydrate intake, glycemic index, or glycemic load) and the risk of bladder cancer were examined by using a random-effects model (24). The possible influence of study design and region on the association between carbohydrate intake, glycemic index, glycemic load, and bladder cancer was investigated by stratified analyses. The impact of each study on the overall OR was investigated by repeating the meta-analysis after omitting each included study in turn.

The linear dose–response trends were computed from the natural logarithm of the ORs and 95% CIs across the categories of exposure (at least three categories) using the method proposed by Greenland and Longnecker (25). In addition, a likelihood ratio test was used to assess non-linearity. For studies that did not provide cases or person-years/non-cases in each category, the total numbers were divided by the number of quantiles. Means or medians of exposure for each category were extracted from the original studies. When only the range of the category was available, the midpoint between the lower and the upper limits was calculated. When a category was open-ended, it was assumed that the range was the same as that of the nearest category.

Heterogeneity between studies was explored using *Q* and *I*^2^ statistics, with the significant level set at *P* ≤ 0.1 (26). Publication bias was evaluated visually by checking funnel plots for asymmetry and by applying Begg's test (27) and Egger's test (28). A two-tailed *P* < 0.05 was considered as statistically significant. All analyses were performed using Stata 11.0 software (StataCorp, College Station, TX, United States).

## Results

### Literature Search and Study Information

**Figure 1:**
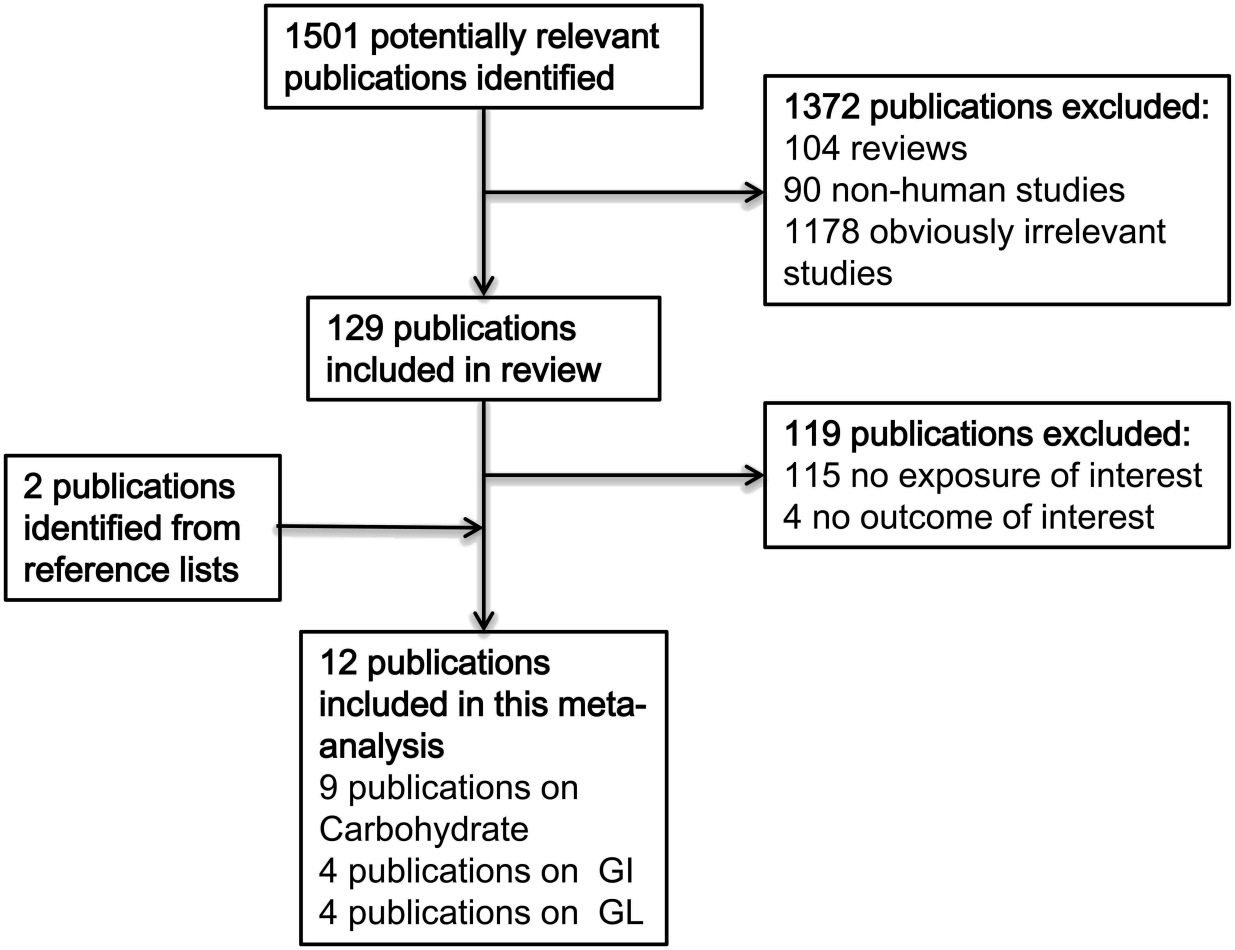
Process of literature search and study selection.

The process of literature search and selection is presented in Figure 1. Twelve studies (11–14, 16–23) on intake of carbohydrates, glycemic index, or glycemic load, and bladder cancer risk were identified. Of these, five were from North America, six were from Europe, and one was from Asia. There were five cohort studies and seven case–control studies. The detailed information of included studies is summarized in Table 1. The study quality was evaluated with NOS, and the scores ranged from 5 to 8, with a mean value of 6.83 (Supplementary Table 2).

### Carbohydrates and Bladder Cancer Risk

**Figure 2:**
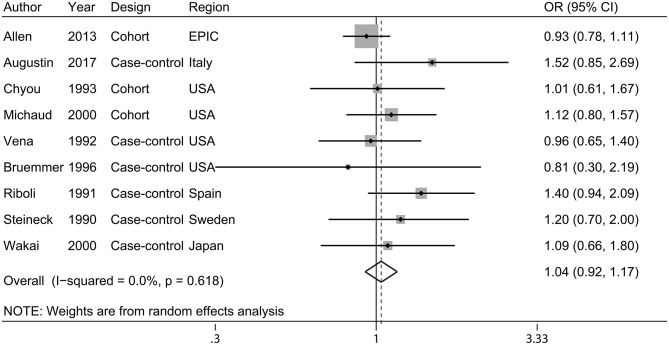
A forest plot showing risk estimates of the association between carbohydrates and bladder cancer risk.

Overall, nine studies (11, 16–23) were included in the meta-analysis on dietary carbohydrate and bladder cancer risk, including 4,071 cases and 530,819 participants. There was no evidence of association between consumption of carbohydrates and bladder cancer risk (pooled OR, 1.04; 95% CI, 0.92–1.17; Figure 2). No statistically significant heterogeneity was detected among the studies (*I*^2^ = 0.0% and *P*_heterogeneity_ = 0.618). In the subgroup analyses, neither geographic region nor study design modified the association between carbohydrate intake and bladder cancer risk (Table 2). Leave-one-out sensitivity analyses showed no marked difference in results, indicating that the summary ORs were not driven by any single study (Supplementary Figure 1). There was no evidence of publication bias (Egger's test: *P* = 0.315; Begg's test: *P* = 0.602, Supplementary Figure 2).

**Table 2:** Subgroup analysis of the association between dietary carbohydrate, GI, and GL, and the risk of bladder cancer.

	**Carbohydrate**				**GI**				**GL**			
	**No. of studies**	**OR (95% CI)**	***p-*value**	**Heterogeneity (p/I^**2**^)**	**No. of studies**	**OR (95% CI)**	***p*-value**	**Heterogeneity (p/I^**2**^)**	**No. of studies**	**OR (95% CI)**	***p*-value**	**Heterogeneity (p/I^**2**^)**
**Study design**												
Cohort	3	0.97 (0.84–1.13)	0.705	0.624/0.0%	2	1.24 (1.02–1.49)	0.067	0.202/37.4%	2	1.01 (0.78–1.32)	0.920	0.827/0.0%
Case–control	6	1.17 (0.96–1.43)	0.126	0.669/0.0%	2	1.24 (0.99–1.56)	0.027	0.757/0.0%	2	1.30 (0.62–2.72)	0.488	0.013/83.8%
**Study region**												
North America	4	1.03 (0.89–1.50)	0.813	0.897/0.0%	2	1.23 (1.06–1.42)	0.007	0.314/13.7%	2	0.95 (0.77–1.16)	0.596	0.933/0.0%
Europe	4	1.15 (0.82–1.28)	0.284	0.132/46.6%	2	1.31 (1.01–1.71)	0.043	0.363/0.0%	2	1.53 (0.92–2.54)	0.098	0.189/42.0%

*GI, glycemic index; GL, glycemic load; No., number; OR, odds ratio; CI, confidence interval*.

### Glycemic Load and Bladder Cancer Risk

**Figure 3:**
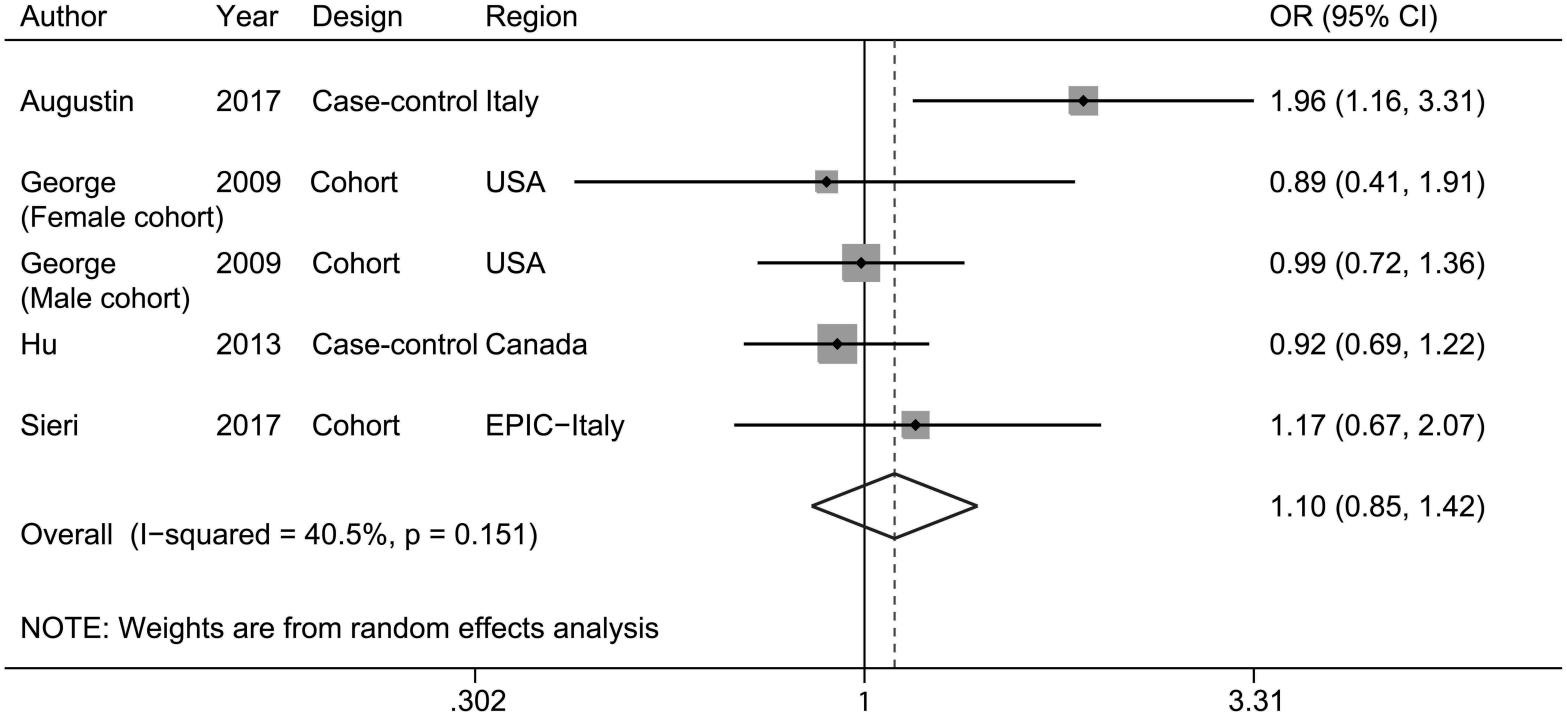
A forest plot showing risk estimates of the association between glycemic load and bladder cancer risk.

Four studies (11–14) investigated the association between glycemic load and bladder cancer risk, with a total of 3,238 cases and 498,895 participants. Overall, no statistically significant association between glycemic load and bladder cancer was found (pooled OR, 1.10; 95% CI, 0.85–1.42; Figure 3). Heterogeneity between the studies was modest (*I*^2^ = 40.5% and *P*_heterogeneity_ = 0.151). There was no evidence of differences by geographic area and study design (Table 2). Leave-one-out analyses showed that the summary results were stable (Supplementary Figure 3).

### Glycemic Index and Bladder Cancer Risk

**Figure 4:**
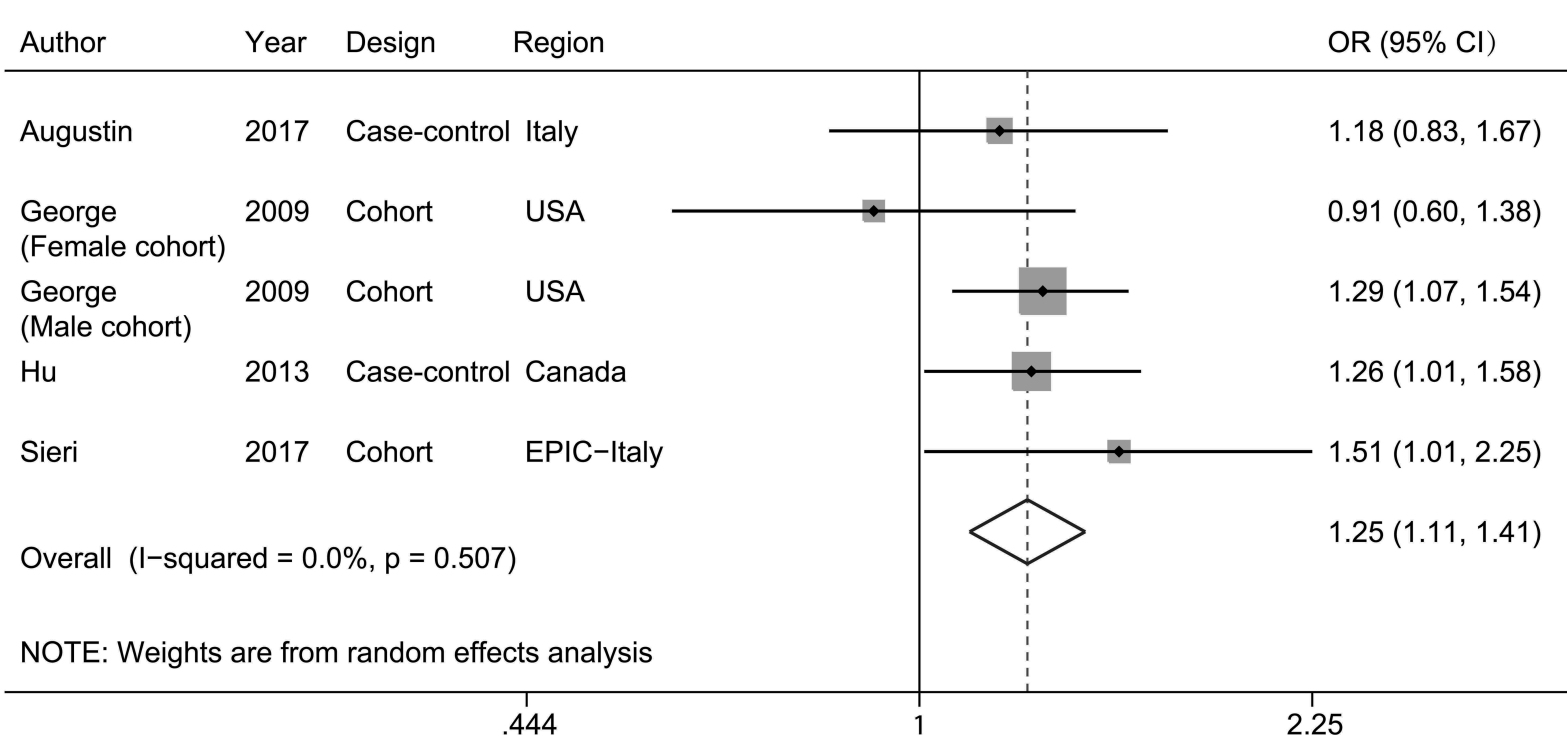
A forest plot showing risk estimates of the association between glycemic index and bladder cancer risk.

Four studies (11–14) were eligible for meta-analysis of dietary glycemic index and bladder cancer risk, with 3,238 cases and 498,895 participants. There was a significant positive association between glycemic index and bladder cancer risk (pooled OR, 1.25; 95% CI, 1.11–1.41; Figure 4), with no evident heterogeneity (*I*^2^ =0.0%; *P*_heterogeneity_ = 0.507). No difference was observed when data were stratified by geographic area and study design (Table 2). Leave-one-out analyses showed that the exclusion of any single study would not lead to the loss of significance (Supplementary Figure 4).

**Figure 5:**
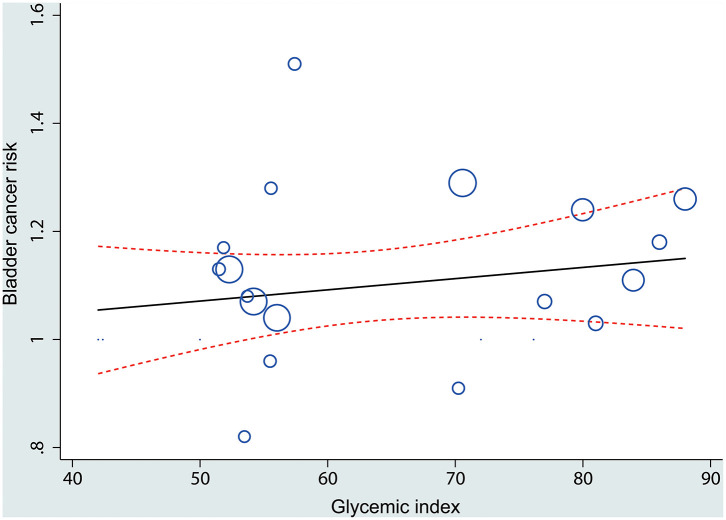
A linear dose–response association between glycemic index and bladder cancer risk. The solid line represents the estimated odds ratios (ORs), and the dotted lines represent the 95% confidence intervals. Circles present the dose-specific ORs reported in each study. The area of each circle is proportional to the inverse variance of the OR.

We then further explored if there was a dose–response relation between dietary glycemic index and bladder cancer risk. There was no evidence of a non-linear association between glycemic index and bladder cancer risk (*P*_nonlinearity_ = 0.389). The curve showed a significant increase in bladder cancer risk with increasing units of glycemic index. The pooled OR (95% CI) per 10 units of glycemic index per day was 1.02 (95% CI, 1.01–1.04) (Figure 5).

## Discussion

In this meta-analysis of observational studies, we found that a higher glycemic index was significantly related to a greater risk of bladder cancer. No association was observed between risk of bladder cancer and carbohydrate intake or glycemic load. To the best of our knowledge, this is the first meta-analysis to evaluate the association between dietary carbohydrates, glycemic index, or glycemic load and bladder cancer risk.

Recently, the relationship between carbohydrates, glycemic index, or glycemic load and the risk of human cancers has been widely studied. Schlesinger et al. (29) and Mullie et al. (30) reported a modest association between high glycemic index or glycemic load and the risk of breast cancer based on meta-analysis of prospective cohort studies. By contrast, there was no evidence of significant association between glycemic index or glycemic load and the risk of prostate cancer (31), colorectal cancer (32), or pancreatic cancer (33). Therefore, the predictive role of glycemic index or glycemic load is not consistent in various human malignancies.

Our study indicated that there was a positive linear association between glycemic index and bladder cancer risk. Glycemic index is an indicator of the quality of carbohydrate foods. Foods with high glycemic index are rapidly digested and lead to a rapid peak in blood glucose and the secretion of insulin (34). Hyperinsulinemia can increase the bioactivity of insulin-like growth factor 1 (35), which may potentially mediate the association between glycemic index and bladder cancer risk. Insulin-like growth factor-1 was able to promote the p-cresidine-induced bladder tumor development in transgenic mice (36). Obesity (6) and diabetes (5), characterized with high IGF-I levels, also have been reported to be positively associated with an increased risk of bladder cancer. However, a recent study nested within the European Prospective Investigation into Cancer and Nutrition cohort failed to find any association between plasma insulin-like growth factor 1 concentrations and bladder cancer risk (37). Therefore, the specific mechanisms mediating the relationship between glycemic index and bladder cancer risk remain unclear.

This meta-analysis has several strengths. First, both high- and low-level analyses and dose–response analysis were performed for the association between glycemic index and bladder cancer risk, with consistent results. Second, there was no evidence of obvious heterogeneity across studies. Third, we performed a throughout literature search, and no publication bias was detected. Fourth, leave-one-out sensitivity analyses showed no marked difference in results, indicating that the pooled risk estimates were robust and not driven by any single study.

As with any study, this meta-analysis also has some limitations. First, individuals with a diet of high carbohydrate intake, glycemic index, or glycemic load may also have other unhealthy behavioral and dietary habits and metabolic-related illness, such as lack of physical activity, excess intake of total energy, diabetes, and obesity, which may confound the true association. Second, measurement error of diet may exist. Food frequency questionnaires are not originally designed to assess glycemic index and glycemic load. Most dietary questionnaires have calculated the glycemic index or glycemic load values according to a limited number of food items, which may affect the accuracy of exposure assessment. Finally, the inclusion of case–control studies may lead to recall and selection bias.

In conclusion, in this meta-analysis, glycemic index showed a positive linear association with bladder cancer risk. Carbohydrate intake and glycemic load were not associated with the risk of bladder cancer. Furthermore, large prospective studies that account for other relevant factors, such as body weight, diabetes, physical activity, and intake of total energy, are needed.

## Data Availability Statement

The datasets generated for this study are available on request to the corresponding author.

## Author Contributions

XX and BL contributed to the conception or design of the work. HZ, QM, and HS contributed to the acquisition, analysis, or interpretation of data for the work. XX and SW drafted the manuscript. HZ and BL critically revised the manuscript. All authors gave final approval and agree to be accountable for all aspects of work ensuring integrity and accuracy.

## Conflict of Interest

The authors declare that the research was conducted in the absence of any commercial or financial relationships that could be construed as a potential conflict of interest. The handling editor declared a shared affiliation with the authors at time of review.

## Supplementary Material

Supplementary Figure 1 Sensitivity analysis was performed for the association between carbohydrates and bladder cancer risk.

Supplementary Figure 2 Publication bias was assessed with Begg's funnel plot.

Supplementary Figure 3 Sensitivity analysis was performed for the association between glycemic load and bladder cancer risk.

Supplementary Figure 4 Sensitivity analysis was performed for the association between glycemic index and bladder cancer risk.

Supplementary Table 1 PICOS criteria for inclusion and exclusion of studies.

Supplementary Table 2 NOS scores of each included study.
