# Supplementary figures and images for: Carbohydrates, Glycemic Index, and Glycemic Load in Relation to Bladder Cancer Risk
Source: Front Oncol. 2020 Sep 23;10:530382. doi: 10.3389/fonc.2020.530382 (PMC7538710; doi:10.3389/fonc.2020.530382)

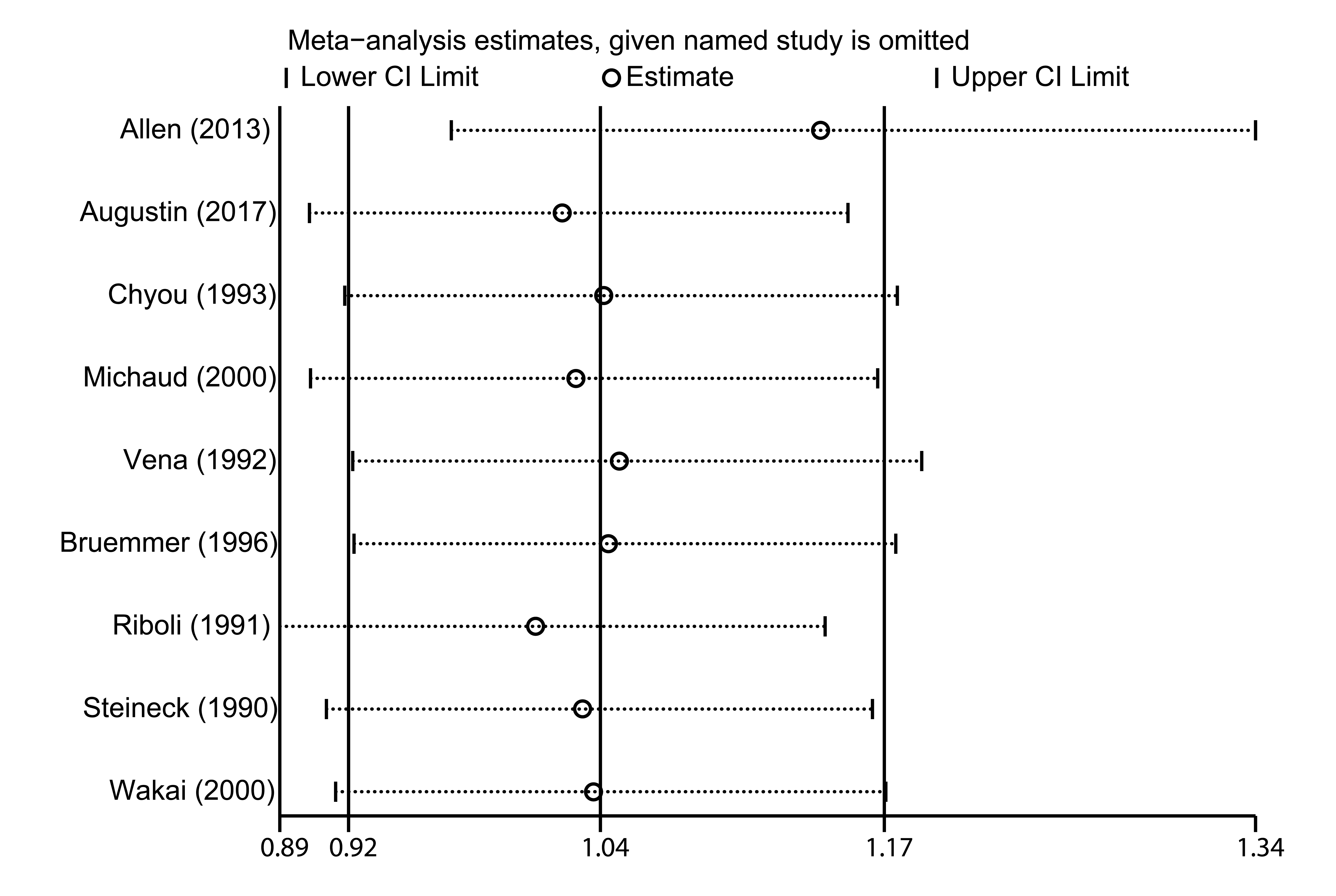

Supplement: Supplementary Figure 1 — Sensitivity analysis was performed for the association between carbohydrates and bladder cancer risk. [file Image_1.TIF]

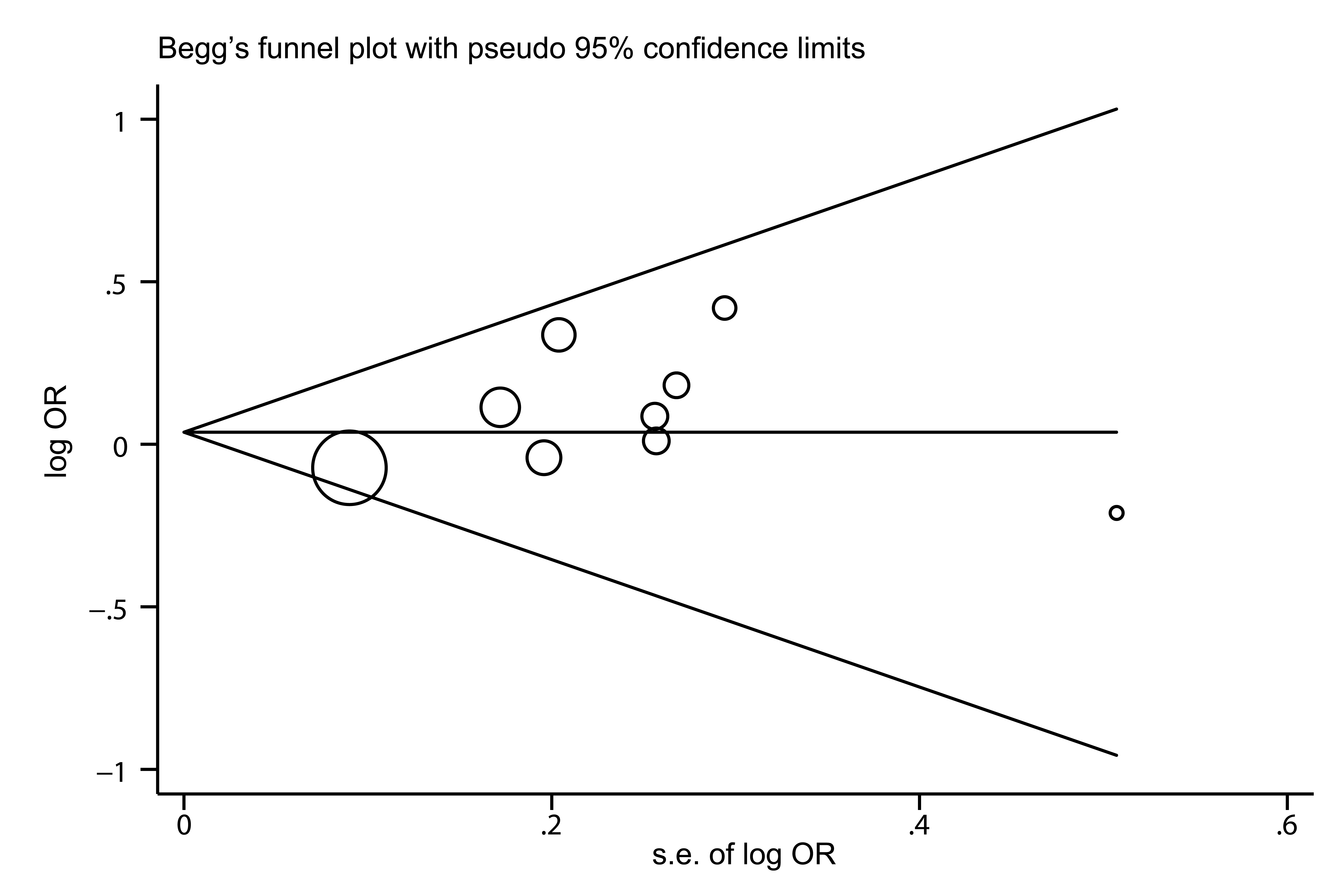

Supplement: Supplementary Figure 2 — Publication bias was assessed with Begg's funnel plot. [file Image_2.TIF]

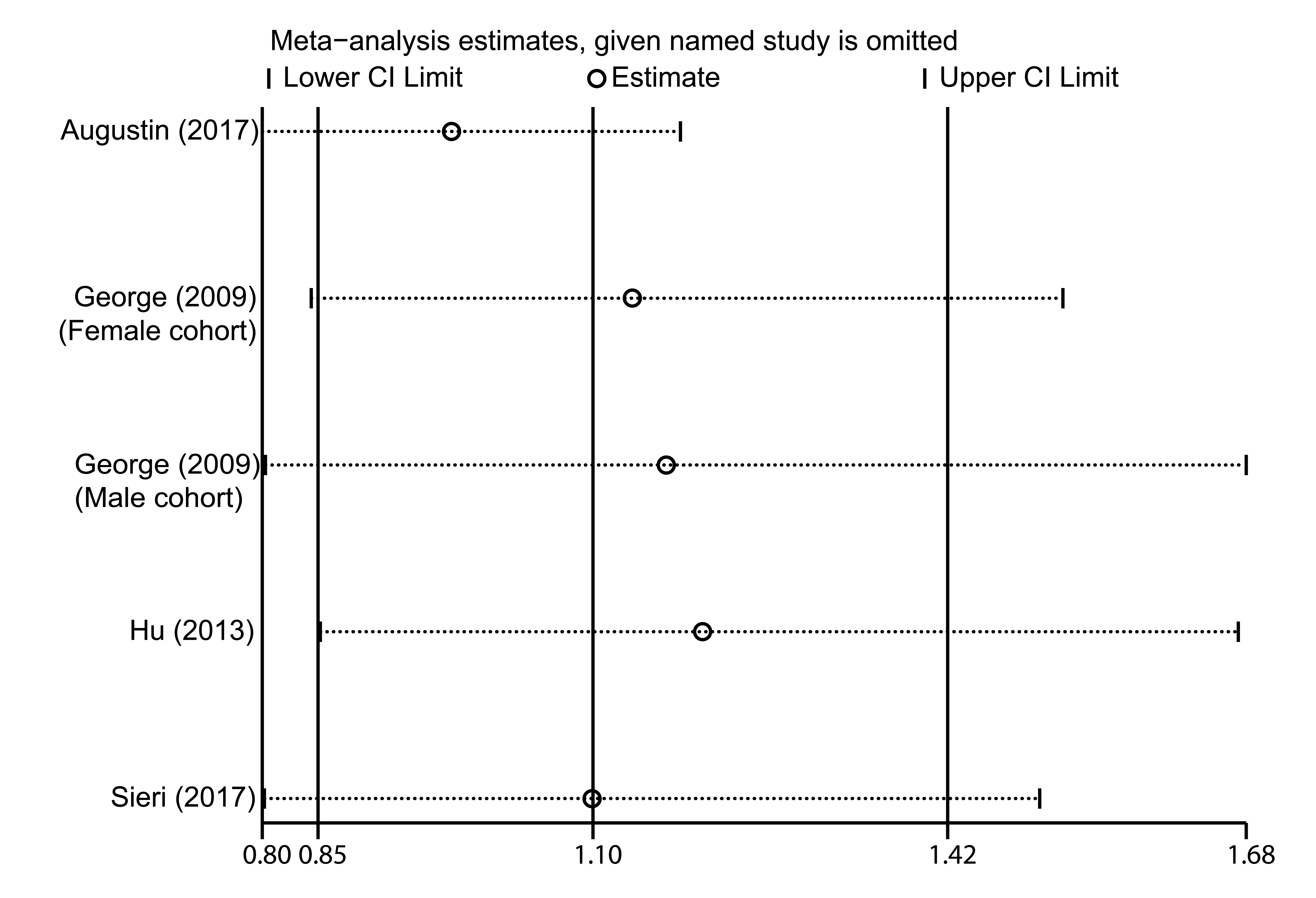

Supplement: Supplementary Figure 3 — Sensitivity analysis was performed for the association between glycemic load and bladder cancer risk. [file Image_3.TIF]

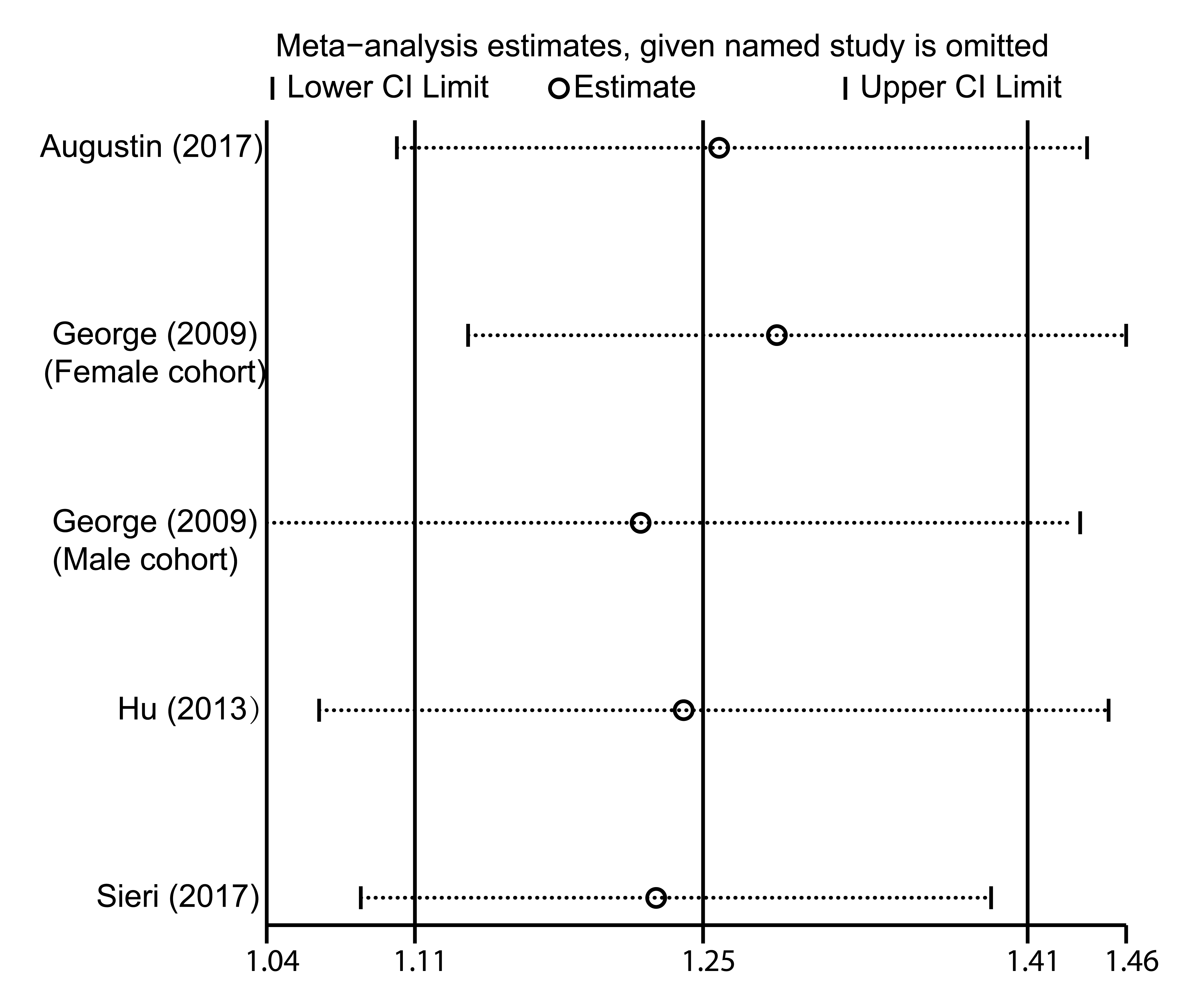

Supplement: Supplementary Figure 4 — Sensitivity analysis was performed for the association between glycemic index and bladder cancer risk. [file Image_4.TIF]
