# Supplementary material for: Carbohydrates, Glycemic Index, and Glycemic Load in Relation to Bladder Cancer Risk
Source: Front Oncol. 2020 Sep 23;10:530382. doi: 10.3389/fonc.2020.530382 (PMC7538710; doi:10.3389/fonc.2020.530382)
Supplement: Supplementary Table 1 — PICOS criteria for inclusion and exclusion of studies. [file Table_1.DOCX]

| **Table S1 PICOS criteria for inclusion and exclusion of studies** | |
| --- | --- |
| **Parameter** | **Inclusion criteria** |
| Population | Women without bladder cancer at baseline |
| Intervention/  exposures | Dietary intake of carbohydrates; glycemic index; glycemic load |
| Comparison | Dose-response relation |
| Outcomes | Bladder cancer |
| Type of study | case-control, nested case-control, cohort and case-cohort studies, and follow-up studies of randomized clinical trials |
