# Supplementary material for: Carbohydrates, Glycemic Index, and Glycemic Load in Relation to Bladder Cancer Risk
Source: Front Oncol. 2020 Sep 23;10:530382. doi: 10.3389/fonc.2020.530382 (PMC7538710; doi:10.3389/fonc.2020.530382)
Supplement: Supplementary Table 2 — NOS scores of each included study. [file Table_2.DOCX]

**Table S2**. NOS scores of each included study

| Case-control study^1^ | Adequate definition of cases | Representativeness  of cases | Selection  of controls | Definition of  controls | Comparability ^2^ | Exposure  assessment | Same method of ascertainment | Non-response  rate^3^ | Total  scores |
| --- | --- | --- | --- | --- | --- | --- | --- | --- | --- |
| Augustin 2017 | * | * |  | * | ** | * | * | * | 8 |
| Hu 2013 | * | * | * | * | * |  | * | * | 7 |
| Vena 1992 | * | * | * |  | * | * | * |  | 6 |
| Bruemmer 1996 | * | * | * | * | * |  | * | * | 7 |
| Riboli 1991 | * | * | * | * | * | * | * | * | 8 |
| Steineck 1990 | * | * | * |  |  | * | * |  | 5 |
| Wakai 2000 | * | * |  | * | * | * | * |  | 6 |
| Cohort study^1^ | Representativeness of the exposed cohort | Selection of the  unexposed  cohort | Ascertainment  of exposure | Outcome of  interest not  present at  start of study | Comparability ^2^ | Outcome  assessment | Follow-up long enough for outcomes to occur ^4^ | Adequacy  of follow-up  of cohorts ^5^ | Total  scores |
| Sieri 2017 | * | * |  | * | ** | * | * | * | 8 |
| Chyou 1993 | * | * | * |  |  | * | * | * | 6 |
| Michaud 2000 | * | * |  | * | * | * | * | * | 7 |
| George 2009 | * | * |  | * | ** | * |  | * | 7 |
| Allen 2013 | * | * |  | * | ** | * |  | * | 7 |

^1^ A study can be awarded a maximum of one star for each item except the item ‘Comparability’.

^2^ A maximum of two stars can be awarded for this item. Studies controlling for or matching by age, smoking, and total energy intake received one star while studies additionally controlling for other important confounders received an additional star.

^3^ One star was assigned if there was no statistically significant difference in the response rate between controls and cases using a chi-square test (*P* > 0.05).

^4^ A cohort study with a follow-up time more than 10 years was assigned one star.

^5^ A cohort study with a follow-up rate more than 75% was assigned one star.
